# Evolutionary Echoes: A Four-Day Fasting and Low-Caloric Intake Study on Autonomic Modulation and Physiological Adaptations in Humans

**DOI:** 10.3390/life14040456

**Published:** 2024-03-29

**Authors:** Pedro Belinchón-deMiguel, Eduardo Navarro-Jiménez, Carmen Cecilia Laborde-Cárdenas, Vicente Javier Clemente-Suárez

**Affiliations:** 1 Department of Nursing, Faculty of Sport Sciences and Physiotherapy, Universidad Europea de Madrid, 28670 Villaviciosa de Odón, Spain; pedro.belinchon@universidadeuropea.es; 2 Grupo de Investigacion en Microbiología y Biotecnología (IMB), Universidad Libre, Barranquilla 080003, Colombia; eduardoi.navarroj@unilibre.edu.co; 3 Vicerrectoría de Investigación e Innovación, Universidad Simón Bolívar, Barranquilla 080005, Colombia; cacelaca6@gmail.com; 4 Grupo de Investigación en Cultura, Educación y Sociedad, Universidad de la Costa, Barranquilla 080002, Colombia

**Keywords:** fasting, ultraendurance, autonomic modulation, heart rate variability, hunter–gatherer diet, body composition

## Abstract

This study evaluates the psychophysiological response to a simulated hunter–gatherer endurance task with restricted caloric intake over four days. It assesses changes in body composition, autonomic modulation, and physical and cognitive performance. Participants underwent daily 8 h fasted walks followed by a 150 kcal meal to replicate hunter–gatherer activity and dietary patterns. Measurements of metabolic, respiratory, and subjective well-being, along with heart rate variability (HRV) monitoring, were conducted pre- and post-activity to evaluate the impact of endurance activity under caloric restriction. We found weight loss, decreased body and visceral fat, and reduced skeletal muscle mass and water percentage. High sympathetic activation and stable urinary markers, except for increased proteinuria, indicated stress responses and muscular degradation. Elevated perceived exertion post-exercise with good adaptation to prolonged effort underlines the body’s adaptability to ancestral lifestyle conditions, highlighting the connection among endurance, nutrition, and psychophysiological health.

## 1. Introduction

Physical activity has been essential in the development of the human species [1]. From our early ancestors, who needed to move and hunt to survive, to modern humans, who sit for extended periods and rely on technology for many tasks, physical activity has been central to human health and well-being. For millions of years, our ancestors lived as hunter–gatherers, relying on physical activity for survival. They needed to run, walk, climb, and carry heavy loads to find food and water, avoid predators, and protect their families [2]. Over time, this physical activity shaped the human body in many ways. For example, walking on two legs, or bipedalism, is a key feature of human evolution that is believed to have developed in part due to the need to travel long distances while foraging for food [3,4].

Despite physical activity being integral to human evolution, contemporary society witnesses a growing trend towards sedentarism among modern individuals. This shift is largely attributed to technological advancements and the dwindling prevalence of physically demanding occupations, resulting in a significant portion of the population spending prolonged periods seated. Such sedentary lifestyles have been associated with various health issues, encompassing obesity, cardiovascular ailments, diabetes, and specific forms of cancer [5]. Moreover, apart from physical health implications, insufficient physical activity has been correlated with mental health challenges, notably depression and anxiety [6]. To address these concerns, the World Health Organization (WHO) advocates for adults to partake in no less than 150 min of moderate-intensity aerobic activity or 75 min of vigorous-intensity aerobic activity per week (World Health Organization, 2021). Despite these recommendations, adherence remains suboptimal. In the United States, for instance, a mere 23% of adults fulfill the suggested levels of physical activity [7].

Another important fact that modulates human evolution is nutritional patterns. The average calorie consumption of hominids throughout our evolution depended on various factors, such as the type and amount of available food, body size, energy expenditure, and morphological adaptations. It is estimated that early hominids, such as Australopithecines, consumed around 2300 calories per day [8]. This calorie consumption was not uniform, and the fasting periods of early hominids depended on the availability and seasonality of food resources, as well as their ability to store and transport food. Australopithecines primarily consumed fruits, leaves, and seeds, which could become scarce during dry or cold periods [9]. The first Homo, who started eating meat and marrow, had more options for obtaining food in different environments and climates but also faced competition and the risk of predation [10]. It is believed that some hominids practiced intermittent fasting or irregular food consumption as an adaptive strategy to survive environmental fluctuations and optimize energy use [11].

In addition to physical effects, physical activity has been shown to significantly impact various cognitive and psychological functions. Recent research has explored how different forms and levels of physical activity can influence cognition, autonomic modulation, and physical performance. For instance, a study by McMorris et al. (2011) found that moderate-intensity aerobic exercise can enhance certain cognitive tasks, such as attention and processing speed [12]. Similarly, research by Dishman et al. (2000) suggests that regular physical activity can improve autonomic modulation, potentially reducing the risk of cardiovascular diseases and enhancing stress responses [13]. In this context, the autonomic nervous system plays a pivotal role in weight control in obesity-related diseases. Indeed, specific neuropeptides and neuronal hormones, such as orexins and adiponectin, contribute to the regulation of locomotor activity, food intake, and metabolic rate [14]. In this line, Polito et al. assessed how a very low-calorie ketogenic diet positively impacts both anthropometric and metabolic levels in obese subjects as well as the activity of the autonomic nervous system. Indeed, heart rate variability (HRV) along with salivary amylase have proven their worth in examining the advancements of dietary therapy [15].

In the context of physical performance, regular physical activity significantly enhances muscle strength, endurance, energy utilization efficiency, and cognitive function and reduces the risk of cognitive impairment, including Alzheimer’s or anxiety and depression diseases [6,16,17,18]. These studies highlight the complex interplay between physical activity and the human body’s physiological and cognitive systems, underscoring the importance of a holistic approach in human health and well-being research.

The objective of this study is to evaluate the psychophysiological response to a simulated hunter–gatherer endurance and restricted caloric intake task over a four-day period. Specifically, the study aims to assess changes in body composition, autonomic modulation, selected physiological and cognitive, and physical performance providing insights into the resilience and adaptability of the human body under conditions mimicking those experienced by early hunter–gatherers. We hypothesize that exposure to a simulated hunter–gatherer endurance task and restricted caloric intake over four days will result in improved body composition (reduced body fat and increased lean muscle mass), enhanced autonomic modulation, improved cognitive function, and enhanced physical performance compared to a control group. These changes are expected to reflect the adaptability and resilience of the human body to conditions resembling those of early hunter–gatherer lifestyles.

## 2. Materials and Methods

### 2.1. Participants

We examined two male volunteers, participant 1, aged 39, standing at 174 cm tall, weighing 72.2 kg, with a body mass index (BMI) of 23.8 kg/m^2^. He engaged in training five days weekly, with an average of one hour per day, comprising three strength training sessions per week utilizing loads between 70% and 80% of his one-repetition maximum (1RM) and two days of running. Running sessions involved continuous work at 60% of maximum heart rate (HR max) alongside varied interval work ranging from 50% to 95% of HR max. Participant 2, aged 37, was 175 cm tall, weighing 80.7 kg, with a BMI of 26.4 kg/m^2^, and followed a similar training regimen, exercising four days per week for an average of one hour daily. This regimen included three days of strength training at 70–80% of 1RM and two days of running, combining continuous and interval work as described. Before the study commenced, participants were fully briefed on the experimental procedures and provided voluntary informed written consent in accordance with the Declaration of Helsinki. The research procedures were designed and approved by the University’s Ethics Committee (CI-PI/22.181).

Given the challenges associated with recruiting participants for extreme events, a convenience sampling method was employed, resulting in the selection of only two participants based on their availability, willingness, and ability to partake in the high-intensity activities required for the research. This non-random selection was deemed necessary due to the unique nature of the events being studied and the difficulty in finding suitable participants with the requisite experience and resilience for such intense scenarios. While convenience sampling facilitated feasibility for this unique study, it is important to acknowledge that results may not be generalizable due to potential biases associated with this sampling method. Nevertheless, the insights gained from these participants offer a valuable starting point and can inform future research in this specialized area.

To ensure the validity and safety of our study on recreating the lifestyle of hunter–gatherers, specific inclusion and exclusion criteria were established to select suitable participants while minimizing potential confounders and risks. Inclusion criteria encompassed male participants aged 18–50, reflecting an age range likely engaged in ancestral hunting and gathering activities; possessing moderate to high levels of physical fitness capable of enduring a four-day hike; devoid of chronic illnesses, cardiovascular issues, or medical conditions that could compromise safety or study outcomes; without strict dietary restrictions or specific allergies; willing to consent to potential caloric restriction and physical demands of the four-day event; and lacking prior participation in extended fasting or extreme physical activities within the past three months. Exclusion criteria included individuals with medical conditions or medications affecting metabolic rates or physiological responses to physical activity and caloric restriction; recent muscle, bone, or joint injuries; strict diets due to medical conditions or personal choices; active smokers or history of substance abuse; known psychological disorders or conditions influencing stress responses; and participation in related research studies within the past three months. These criteria were formulated to ensure selected participants were representative of a typical healthy individual from ancestral times while avoiding confounding factors in line with previous research in this domain [19].

### 2.2. Ultraendurance Probe

Table 1 provides a comprehensive overview of the ultraendurance probe conducted in the study. It detail, the distance covered in kilometers, duration in hours and minutes, as well as the elevation statistics, including positive elevation, negative elevation, and cumulative elevation in meters, are reported for each of the four days of the probe. Additionally, the table presents the maximum and minimum temperature (in °C) recorded during the four-day period. The “Total” row summarizes the cumulative values for the entire probe, highlighting the total distance, duration, and elevation gain over the course of the endurance task. These data points serve as essential parameters for understanding the physical challenges and environmental conditions participants encountered during the ultraendurance probe.

Throughout each day of the ultraendurance probe, participants consumed a daily ration of 40 g of an energy bar with the composition as depicted in Table 2.

### 2.3. Experimental Procedures

The study aimed to investigate the psychophysiological responses to a four-day simulated hunter–gatherer endurance task with restricted caloric intake. To provide a comprehensive understanding of these responses, baseline measurements were initially collected, encompassing body composition, autonomic modulation, physiological and cognitive parameters, and physical performance. The core of the study involved a daily eight-hour walking activity, mimicking the physical demands of a hunter–gatherer lifestyle, commencing at 08:00 AM. Participants’ caloric intake during these four days was limited to 150 kilocalories in the afternoon, simulating the dietary challenges faced by early hunter–gatherers, as employed in previous studies [19]. Post-activity assessments were conducted to monitor changes in body composition, autonomic modulation, physiological and cognitive parameters, and physical performance. These evaluations allowed for an in-depth analysis of the body’s response to the endurance task and caloric restriction. Subsequently, a three-day recovery period was observed, during which participants had ad libitum access to food, and body composition was analyzed to observe the body’s adaptability and resilience post-endurance task.

### 2.4. Study Variables

Before and after each day of the event, we assessed the following parameters in accordance with prior research [19,20,21,22,23,24]:

- Body Composition: Measurements were taken using the validated InBody 270 bioelectrical impedance analysis (BIA) device, which is renowned for its accuracy and reliability in assessing body composition. This device employs a multi-frequency, segmental BIA method, utilizing eight-point tactile electrodes to provide detailed body composition readings. Participants stood on the device’s platform with their legs slightly apart and arms not touching the torso, in a similar posture required for the previously mentioned device. They were barefoot and wore minimal clothing to ensure precise measurements. The InBody 270’s electrodes, designed to optimize contact and improve the accuracy of the measurement, required no additional preparation of the skin. Participants were instructed to grasp the hand electrodes according to the InBody’s specific protocols [25]. The utilization of the InBody 270 was justified due to its validated methodology for providing precise and segmental body composition analysis, including muscle mass, fat mass, and water distribution. This validated device is widely recognized for its clinical accuracy and reproducibility, making it an ideal choice for our study to ensure high-quality, reliable body composition data [26].
- Body Mass Index (BMI): Calculated as weight in kilograms divided by height in meters squared (kg/m^2^), following World Health Organization guidelines.
- HRV: Monitored using a Polar V800 HRV monitor (Kempele, Finland). Measurements commenced minutes before the event’s onset and concluded upon its completion.
- Reaction Time: Assessed using a web-based test accessed through a mobile device via the URL https://www.arealme.com/reaction-test/es/ (accessed on 27 November 2023). The test interface displayed on the phone’s screen transitions from white to a randomly chosen color, prompting the participant to touch the screen as quickly as possible. Prior to the actual measurements, participants were given an opportunity to familiarize themselves with the procedure. Three measurements were recorded both before and after the intervention. The final reaction time value was determined by calculating the average of these three measurements [27].
- Handgrip Strength: Isometric handgrip strength was measured using a TKK 5402 dynamometer (Takei Scientific Instruments Co. Ltd., Niigata, Japan). The participant’s dominant hand grip strength was evaluated. The participant sat with 0 degrees of shoulder flexion, 90 degrees of elbow flexion, and the forearm in a neutral position. The highest result of two trials was recorded.
- Jump Height: Lower limb strength was assessed using a horizontal jump test. The participant stood behind a marked line on the ground with feet shoulder-width apart. Three attempts were made, and the highest result was recorded.
- Forced Vital Capacity, Forced Expiratory Volume in 1 Second, Peak Expiratory Flow: These parameters were measured using a QM-SP100 spirometer (Quirumed, Valencia, Spain) during a maximum inhalation–exhalation cycle.
- Body Temperature: Measured using a digital infrared thermometer (Temp Touch; Xilas Medical, San Antonio, TX, USA).
- Blood Glucose Levels: Determined by analyzing 5 μL of capillary blood from the finger using a portable analyzer (One Touch Basic, LifeScan Inc., Madrid, Spain).
- Hydration Status: Immediately after the event, hydration status was evaluated using a colorimetry procedure with a urine color chart (UCC), assisting in identifying pH status and the presence of glucose, nitrites, proteins, and urine specific gravity (USG), following established protocols [19,20,21].
- Rate of Perceived Exertion (RPE): Based on the 6 to 20 scale [28].
- Subjective Pain Level, Leg Pain Level, Hunger Level: Each assessed on a self-reported scale from 0 to 100, with 0 being the lowest value and 100 the highest for the level.

## 3. Results and Discussion

The two male volunteers evaluated in this ultraendurance event provided valuable insights into the physiological changes associated with prolonged exercise and caloric deficit (Table 3 and Table 4). These participants’ weight loss is consistent with existing literature, which emphasizes the benefits of combining exercise with a caloric deficit for weight loss. It is important to note that factors like exercise intensity, timing, and the participant’s caloric intake significantly influence the extent of weight loss. A key observation in this study is the timing of exercise activities, which were conducted early in the morning. According to Willis et al. (2020), exercising early in the day may boost weight loss. Their findings show that early exercise leads to more weight loss than late exercise, despite minimal differences in components of energy balance [29]. This aligns with our observations, where participants engaged in over 200 min of activity per day, a duration proven effective for weight loss. Swift et al. (2014) noted that adults exercising more than 200 min weekly experienced greater weight loss than those exercising between 150 and 199 min weekly [30].

Regarding body fat distribution at the start of the trials, we observed a higher percentage of total and segmental body fat in participant 2. However, we found that in both participants, the fat mass decreased when comparing results from the initial analysis to those at the end of the trials on the fourth day. This decrease occurred in both the total percentage of body fat and at a segmental level, especially in the trunk area. Evidence shows how humans can significantly adjust our energy requirements based on nutrient availability. Hence, in carbohydrate-scarce situations, humans have developed an efficient metabolism for using fats as an energy source [31]. Furthermore, it has been demonstrated that even in elite endurance athletes, a ketogenic diet over four weeks does not negatively impact performance [32]. Highly trained ultraendurance athletes who have had sufficient time to adapt their metabolism to a ketogenic diet show great efficiency in conserving glycogen and oxidizing fats at various intensities [33]. Consequently, in the situation of caloric restriction with a high energy demand, the participants in this study were supported by their evolutionary metabolism, which aids in burning lipids as fuel during scarcity [34].

Regarding visceral fat, a comparison between initial results and those obtained at the end of the fourth day showed a decrease in visceral fat levels for both participants: from level 5 to level 3 in participant 1 and from level 8 to level 6 in participant 2. The literature underscores the significance of visceral fat in current population health, identifying it as an independent risk marker for both cardiovascular and metabolic morbidity and mortality. It is also a risk factor for type 2 diabetes, atherosclerosis, and cardiovascular diseases [35]. Ismail et al., in their 2012 meta-analysis, established that aerobic exercise is crucial in reducing visceral fat, as well as in addressing overweight and obesity issues [36]. Therefore, the health benefits of prescribing aerobic exercise combined with advice to moderate caloric intake to meet the actual needs of the population should be considered urgent measures to implement in public health [37].

Moreover, it is noteworthy that on the third day post-ultraendurance event, both participants showed an increase in their visceral fat levels, concluding the measurements at levels 4 and 7, respectively. Evidence indicates that diet combined with exercise is more effective than either alone. Additionally, exercise reduces the “yo-yo” effect often seen with diets [38]. Our research observed a rapid regain of visceral fat in the participants once caloric restriction and high physical activity ceased. Therefore, it is crucial to maintain habits more aligned with our evolutionary heritage, as opposed to those of modern civilization, which promote sedentarism and the consumption of hypercaloric foods with little nutritional value [39].

Regarding skeletal muscle mass, we found a decrease in both participants when comparing results from the initial analysis to those at the end of the fourth day. However, values higher than those found on the first day were recorded on the second day post-event. Ultraendurance events typically induce a range of physiological responses, including muscle damage and fatigue, due to the significant stress they place on the body [40]. This stress leads to increased energy expenditure and breakdown of muscle tissue, although the specific degree of muscle mass loss varies based on individual fitness levels, training status, nutritional habits, and recovery strategies [41]. Muscle mass loss in these contexts is often attributed to muscle protein breakdown, inflammatory responses, and elevated cortisol levels. This combination of factors can lead to temporary muscle damage, requiring muscle protein synthesis for repair and rebuilding [42]. Studies have consistently shown that ultraendurance events lead to muscle mass loss; however, the extent of this loss can vary widely among individuals and across different events [43]. The muscle damage is often a result of metabolic overload and/or mechanical strain [44].

Regarding total water percentage, we observed a decrease in both participants when comparing the results from the initial analysis to those at the end of the fourth day. This finding contrasts with previous research, where a 52 km ultraendurance race did lead to increases in water percentage. A possible influencing factor is that the current study did not involve running, as in the 52 km event, which likely resulted in less edema [25]. Additionally, caloric restriction in this study might have influenced hydration status. The evidence recommends drinking according to thirst in these types of events and maintaining a usual diet to stay adequately hydrated [45]. Indeed, we found higher water percentage values on the second day after the event compared to the beginning, likely due to increased ad libitum water intake during the days of physical exertion and caloric restriction.

Regarding urinary markers, we did not observe differences in both participants. We also found no glucose or nitrates or an altered urine pH. This aligns with previous research where a multi-stage low-intensity ultraendurance event did not produce alterations in these parameters [19]. The most notable finding is the presence of proteinuria in both participants from the third day. Although protein degradation decreases in fasting situations [46], the presence of proteinuria could be justified by muscular degradation and protein catabolism [47].

**Figure 1:**
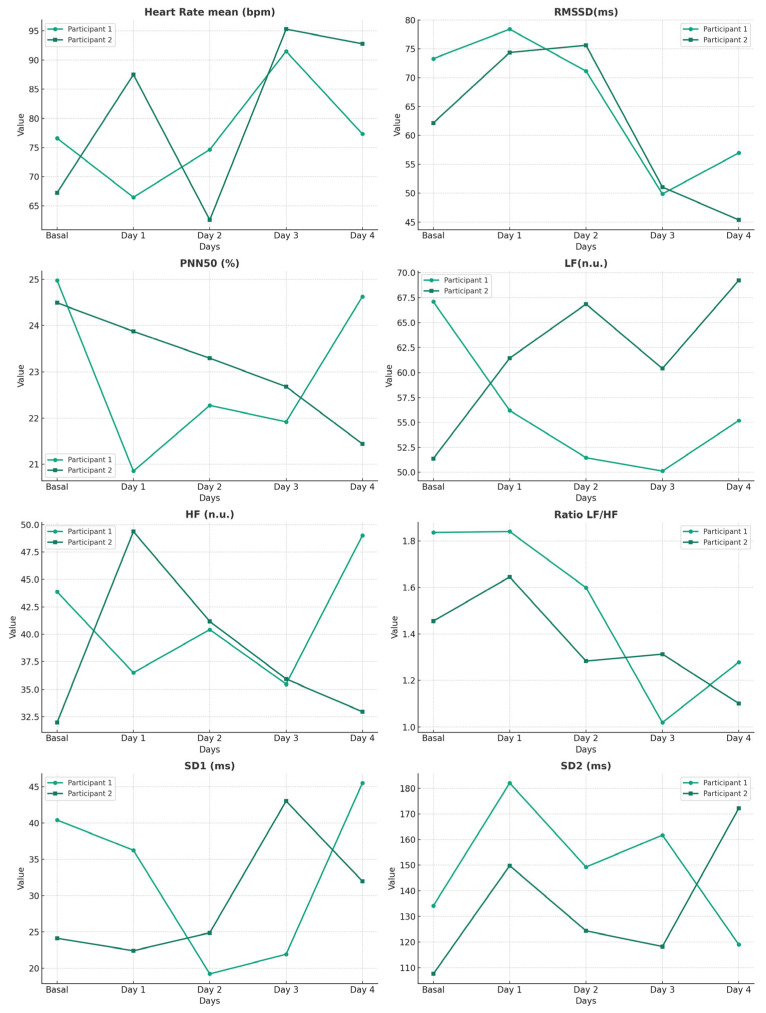
HRV modifications of participants during the ultraendurance event. (HR): heart rate; (Min HR): minimum heart rate; (Max HR): maximum heart rate; (RMSSD): the square root of the average of the sum of the differences squared between normal adjacent R-R intervals; (PNN50): percentage of differences between normal adjacent R-R intervals greater than 50 ms; (LF): the low-frequency band in normalized units; (high-frequency, HFn): the high-frequency band in normalized units; (SD1): sensitivity of the short-term variability; (SD2): sensitivity of the long-term variability.

In terms of autonomic modulation analysis using HRV (Figure 1), we found high sympathetic activation, showing a slight habituation over the days. This finding is consistent with evidence indicating that ultraendurance races are stressful events causing an elevation in sympathetic modulation that is maintained until the end of the test [25]. Additionally, caloric restriction did not affect sympathetic activation. This is in line with previous research on multi-stage ultraendurance tests and fasting [19]. Thus, the results provide new evidence on how ultraendurance events affect sympathetic activation regardless of the participant’s nutritional status. As for cortical activation, no clear trend was observed after each stage in both participants. Specifically, the repercussions of mental fatigue caused by exercise could be a result of the measurement task, timing, exercise duration [48], or the intensity of the test [49]. Consequently, more studies are needed to determine whether mental fatigue is induced by ultraendurance events.

The levels of RPE were elevated in both participants after the completion of each test day. This finding is consistent with the literature, which suggests that finishing an ultraendurance event, even at low intensities, results in high levels of perceived effort [45]. Indeed, caloric restriction in situations of high physical demand has also been implicated in increasing this perception of effort [19]. However, it should be noted that the values prior to the tests remained low and constant over time. This suggests a high level of adaptation by the participants to the repeated prolonged low-intensity efforts day after day, even under conditions of caloric restriction. In fact, reviewed evidence shows how humans have evolved to face these types of challenges thanks to having a high metabolic efficiency for long-distance events [50].

Regarding pain values and leg pain, they gradually increased, reaching maximum values after the third day’s completion. The literature highlights the benefits of increased training volume in relation to higher pain tolerance [51]. In our study, participant 2 presented higher pain levels and less weekly training time than participant 1. However, we understand that while this fact may influence, it would not be the sole cause justifying these differences in perceived pain between both participants. Likely, the higher BMI in participant 2, at 26.4 (indicating slight overweight >25), is also related to greater perceived pain. It is known that in ultraendurance tests, BMI negatively correlates with execution time. Additionally, a lower BMI is a good indicator of successful completion in these types of events [52]. Therefore, this difference with participant 1, along with less weekly training, could explain the data found in pain perception. To cover the same distance, participant 2 presents greater inefficiency in their gait, requiring greater effort. Thus, this circumstance could be related to greater perceived pain and negatively impact performance [53]. Furthermore, we observed a greater sensation of hunger in participant 2. The literature shows how hunger is related to an increase in pain and contributes to the ideation of negative perceptions that can diminish an athlete’s capacity [54]. Consequently, the increase in perceived pain would have a multifactorial explanation, which requires further investigation in the context of ultraendurance tests and caloric restriction. In this regard, in the present study, we found a greater sensation of hunger in participant 2 at all evaluated moments, with much higher differences on the days following the tests. Evidence shows that overweight people, as is the case with participant 2, and those with obesity may have difficulties in correctly interpreting signals related to food intake control due to a presumable decrease in interoceptive sensitivity [55]. Therefore, enhancing control over satiety and the voluntary restriction of food could be key in adapting to situations of scarcity and the need to possess higher metabolic efficiency [11].

Regarding variables related to expiratory lung capacity, we found similar values in both participants, with the particularity of an upward trend until the third day. Literature shows that in ultraendurance mountain tests of up to 65 km, no significant signs of expiratory lung fatigue were shown, contrary to what occurs in tests of distances greater than 100 km [56]. It could be that the activity undertaken by the participants in our study could increase expiratory capacity, producing necessary adaptations to endurance training. Therefore, athletes could benefit from programs adapted to the study of the strength of the expiratory lung musculature without reaching the production of fatigue [56]. In this line, we found a decrease in the strength of the lower limbs at the end of each stage. Beyond the differences between both participants, probably related to the quality of training as well as BMI [24], the athletes accused the typical wear and tear shown in ultraendurance tests where the greatest demand is placed on the strength of the leg muscles [21]. However, this decrease does not present an absolute feature, as significant increases in hand strength have been found as a result of sympathetic activity, in accordance with our research [23].

### 3.1. Practical Applications

This study offers insights into managing physical activity and caloric intake, particularly for ultraendurance activities. It underscores the importance of personalized hydration strategies and the nuanced effects of caloric restriction combined with intense physical exertion. The findings highlight the need for individualized approaches to such activities, considering physiological responses and potential health risks. This research also informs public health policies, emphasizing the balance among exercise, diet, and lifestyle that aligns with human evolutionary adaptations for optimal health and well-being.

### 3.2. Limitation of the Study and Future Research

The main limitation of this study is its small sample size, which may compromise the generalizability of the findings to broader populations. Additionally, the specific participant profiles, including their prior training and nutritional habits, could influence the results. Future research should focus on larger and more diverse populations to validate these findings. Investigations into different durations and intensities of activities, as well as the long-term health impacts of such regimens, are also essential. Further exploration into the role of individual physiological differences in response to extreme physical and nutritional conditions would provide a more comprehensive understanding of the subject.

## 4. Conclusions

This study’s exploration of the psychophysiological responses to a simulated hunter–gatherer endurance task with restricted caloric intake over four days has illuminated the resilience and adaptability of the human body under conditions reminiscent of our ancestral lifestyle. Our findings of weight loss; decreased total and segmental body fat, particularly in the trunk area; and a reduction in visceral fat levels underscore the health-promoting potential of integrating physical activity and dietary modulation reminiscent of hunter–gatherer patterns. Importantly, the observed decrease in skeletal muscle mass and total water percentage, alongside the stability of urinary markers with the exception of proteinuria, signals the physiological cost and adaptation processes associated with such endurance activities. The heightened sympathetic activation measured through HRV further delineates the stress response inherent to ultraendurance events, even in the face of nutritional challenges.

The nuanced outcomes of this study, including the elevated levels of perceived exertion post-exercise that contrast with low prior values, highlight a remarkable adaptation to prolonged, low-intensity efforts under conditions of caloric restriction. These insights contribute to our understanding of human physiological and psychological endurance and adaptability, offering a deeper appreciation of our evolutionary heritage and its implications for modern health and fitness practices.

In light of these findings, we advocate for a more holistic approach to health and fitness that embraces the principles of endurance and nutritional status inherited from our ancestors. Such practices, informed by the intricate relationship among physical activity, dietary patterns, and psychophysiological health, can offer valuable strategies for addressing contemporary health challenges.

### Future Directions

While this study has provided valuable insights, it also underscores the need for further research involving larger and more diverse populations to validate these findings. Future studies should explore the long-term impacts of integrating ancestral lifestyle patterns into modern life, examining the effects on different demographics and within various environmental contexts. Additionally, investigating the role of individual physiological and psychological differences in response to endurance and dietary challenges will enhance our understanding and application of these findings.

By expanding our knowledge in these areas, we can better inform public health policies and personal health practices, fostering a society that not only appreciates but also embodies the resilience and adaptability of our ancestors. Through this, we aim to not only improve individual health outcomes but also address broader societal health challenges, guiding us towards a healthier, more harmonious existence with our evolutionary heritage.

## Figures and Tables

**Table 1:** Description of the ultraendurance event.

	Distance Covered (km; m)	Duration (h; min)	Positive Elevation (m)	Negative Elevation (m)	Cumulative Elevation (m)	Maximum Temperature (°C)	Minimum Temperature (°C)
Day 1	40	8:00	755	745	1500	16	5
Day 2	36.31	7:53	250	205	455	12	3
Day 3	32.94	8:25	405	385	790	14	5
Day 4	30.55	7:53	300	300	600	16	3
Total	139.8	32:11	1710	1635	3345	14.5 medium	4 medium

m: meters, km: kilometers.

**Table 2:** Nutritional composition of daily intake of the participants during the ultraendurance event.

Nutrient	40 g	Per 100 g
Energy (kJ)	628.4 kJ	1571 kJ
Energy (kcal)	151.2 kcal	378 kcal
Lipids (Fats)	7.2 g	18 g
Saturated Fats	4.3 g	10.8 g
Carbohydrates	14.35 g	35.9 g
Sugars	0.2 g	0.47 g
Polyalcohols	12.55 g	31.4 g
Fiber	1.3 g	3.3 g
Proteins	11.2 g	28 g
Salt	0.35 g	0.83 g

g: grams, kJ: kilojoules, kcal: kilocalories. Nutrient represents the nutritional value of the energy bar (40 g), and “Per 100 g” indicates per 100 g of the product.

**Table 3:** Body composition modifications during the study.

Participant	Evaluation Moment	Weight	Total Body Water	Protein	Body Fat Mass	Fat Free Mass	Skeletal Muscle Mass	Body Mass Index	Percent Body Fat	Fat Free Mass of Right Arm	Fat Free Mass of Left Arm	Fat Free Mass of Trunk	Fat Free Mass of Right Leg	Fat Free Mass of Left Leg	Body Fat Mass of Right Arm	Body Fat Mass of Left Arm	Body Fat Mass of Trunk	Body Fat Mass of Right Leg	Body Fat Mass of Left Leg	Visceral Fat Level	Obesity Degree 90–110
1	1	72.2	42.1	11.6	14.6	57.6	32.8	23.8	20.2	3.00	3.01	24.9	9.68	9.45	0.8	0.8	7.2	2.4	2.3	Level 5	108
	2	71.2	42.6	11.7	13.0	58.2	33.3	23.5	18.3	3.11	3.03	25.2	9.84	9.63	0.6	0.7	6.4	2.2	2.1	Level 4	107
	3	70.1	42.1	11.7	12.4	57.7	33.0	23.2	17.7	3.04	2.98	25.0	9.75	9.61	0.6	0.6	6.0	2.1	2.1	Level 4	105
	4	70.1	41.8	11.6	12.8	57.3	32.8	23.2	18.3	2.96	3.02	24.9	9.75	9.50	0.6	0.6	6.2	2.2	2.1	Level 4	105
	5	69.0	41.3	11.3	12.6	56.4	32.3	22.8	18.2	2.91	2.98	24.7	9.67	9.47	0.6	0.6	6.0	2.1	2.1	Level 4	104
	6	68.9	42.1	11.6	11.4	57.5	33.0	22.8	16.5	3.08	3.11	25.4	9.61	9.47	0.5	0.5	5.5	1.9	1.9	Level 3	103
	7	68.1	41.0	11.3	12.0	56.1	32.1	22.5	17.7	2.88	2.96	24.5	9.56	9.38	0.6	0.6	5.8	2.0	2.0	Level 3	102
	8	68.3	41.1	11.2	12.1	56.2	32.1	22.6	17.7	2.89	2.84	24.2	9.55	9.42	0.6	0.6	5.8	2.1	2.0	Level 3	103
	9	67.4	40.6	11.2	11.8	55.6	31.8	22.3	17.5	2.87	2.93	24.5	9.50	9.34	0.6	0.6	5.6	2.0	2.0	Level 3	101
	10	69.4	42.6	11.7	11.1	58.3	33.2	22.9	16.0	2.99	3.04	24.8	9.79	9.66	0.5	0.5	5.3	1.9	1.9	Level 3	104
	11	69.7	41.8	11.4	12.6	57.1	32.5	23.0	18.0	2.93	2.98	24.5	9.74	9.55	0.6	0.6	6.0	2.1	2.1	Level 4	105
2	1	80.7	44.3	12.2	20.1	60.6	34.7	26.4	25.0	3.49	3.56	28.0	9.23	9.32	1.1	1.1	11.2	2.7	2.7	Level 8	120
	2	78.9	43.3	12.0	19.5	59.4	34.1	25.8	24.8	3.32	3.26	26.9	9.38	9.43	1.1	1.1	10.5	2.8	2.8	Level 7	117
	3	77.7	43.2	11.9	18.5	59.2	33.9	25.4	23.9	3.24	3.31	26.7	9.32	9.40	1.0	1.0	9.9	2.7	2.7	Level 7	115
	4	77.0	43.2	12.0	17.8	59.2	34.0	25.1	23.1	3.24	3.26	26.5	9.34	9.44	1.0	1.0	9.4	2.6	2.6	Level 6	114
	5	76.8	42.8	11.8	18.2	58.6	33.6	25.1	23.6	3.21	3.18	26.3	9.41	9.59	1.0	1.0	9.5	2.7	2.7	Level 6	114
	6	76.2	42.6	11.7	17.9	58.3	33.4	24.9	23.5	3.19	3.12	26.1	9.37	9.53	1.0	1.0	9.3	2.7	2.7	Level 6	113
	7	76.3	42.5	11.7	18.1	58.2	33.4	24.9	23.7	3.20	3.16	26.2	9.40	9.64	1.0	1.0	9.5	2.7	2.7	Level 6	113
	8	77.1	43.9	12.1	17.0	60.1	34.3	25.2	22.1	3.24	3.25	26.3	9.65	9.88	0.9	0.9	8.8	2.6	2.6	Level 6	114
	9	77.9	43.7	12.0	18.1	59.8	34.3	25.4	23.2	3.31	3.43	27.2	9.17	9.23	1.0	1.0	9.9	2.5	2.5	Level 7	116
	10	78.5	44.6	12.3	17.4	61.1	34.8	25.6	22.2	3.29	3.29	26.5	9.60	9.92	0.9	1.0	9.1	2.6	2.7	Level 6	117
	11	78.8	43.7	12.0	19.0	59.8	34.3	25.7	24.1	3.40	3.40	27.3	9.21	9.25	1.1	1.1	10.4	2.6	2.6	Level 7	117

Evaluation moment 1: pre day 1; 2: post day 1; 3: pre day 2; 4: post day 2; 5: pre day 3; 6: post day 3; 7: pre day 4; 8: post day 4; 9: day 5; 10: day 6; 11: day 7.

**Table 4:** Psychophysiological parameter modifications during the study.

		1—Pre Day 1		2—Post Day 1		3—Pre Day 2		4—Post Day 2		5—Pre Day 3		6—Post Day 3		7—Pre Day 4		8—Post Day 4		9—Day 5		10—Day 6		11—Day 7	
Participants	Unit	1	2	1	2	1	2	1	2	1	2	1	2	1	2	1	2	1	2	1	2	1	2
Reaction time	(ms)	292	294	317	314	258	310	282	284	309	258	298	289	289	293	283	231	280	259	311	287	262	276
Handgrip	kg	54.8	43.8	50.9	54	54.2	44.4	56.9	51.4	50.1	45.6	54.3	47.6	50.5	46.7	52.5	48.1	51.1	47.7	50.3	43.8	50	42.8
Horizontal Jump	cm	130	135	100	105	115	99	85	67	110	101	92	55	110	80	94	68	110	94	110	90	111	91
FVC		3.78	4.16	3.88	4.08	4.34	4.16	4.14	4.1	4.15	4.19	4.36	4.02	4.11	4.1	4.34	4.12	4.34	4.11	4.12	4.2	4.22	4.7
FEV1		3.29	3.72	3.4	3.47	3.58	3.79	3.63	3.86	3.66	3.75	3.6	3.71	3.55	3.76	3.66	3.79	3.79	3.75	3.64	3.7	3.63	3.7
PEF		11.8	12.43	6.68	12.2	11.88	9.78	11.67	9.85	12.09	11.98	12.31	12.43	11.37	11.37	12.43	12.2	11.98	11.77	12.31	12.31	11.98	12.2
Tª	°C	31.4	29.5	31.2	32.2	31.5	30.9	31.9	31.7	31.2	31.6	32.2	32	31.3	31	32.2	32.2	31.6	30.4	31.9	32.1	31.9	31.5
Glucose	mmol/L	7.7	7.7	5.7	7.7	7.1	6.8	5.1	6.1	7.1	7.6	5.4	6.7	7.3	8.2	5.1	6.8	6.5	7.6	7.4	7.9	7.2	7.2
USG		1.02	1.02	1.01	1.02	1.02	1.03	1.00	1.03	1.01	1.02	1.00	1.03	1.02	1.02	1.00	1.03	1.01	1.02	1.01	1.01	1.02	1.02
UCC		2	2	4	3	3	3	1	6	4	4	2	5	4	5	1	5	4	3	3	3	4	4
Nitrites urine		N	N	N	N	N	N	N	N	N	N	N	N	N	N	N	N	N	N	N	N	N	N
pH urine		6	5	5	5	5	5	5	6	5	5	5	6	5	6	6	6	7	7	6	6	7	6
Protein urine		N	N	N	N	1	1	N	1	1	1	1	1	2	1	2	2	1	2	1	1	1	1
Glucose urine		N	N	N	N	N	N	N	N	N	N	N	N	N	N	N	N	N	N	N	N	N	N
RPE (6–20)		6	6	15	18	8	10	15	16	7	8	18	18	9	11	16	15	9	10	7	10	7	8
Pain (0–100)		0	10	50	30	15	30	50	51	30	40	55	80	30	40	50	65	20	45	20	30	15	30
Pain leg (0–100)		0	10	60	40	25	50	70	65	40	60	65	90	40	60	65	70	30	65	25	40	20	40
Hunger		0	7	5	17	10	25	30	42	15	20	25	30	25	45	20	52	15	40	0	40	0	35

FVC: Forced vital capacity, FEV1: forced expiratory volume in 1 s, PEF: peak expiratory flow, Tª: temperature, USG: urine specific gravity, UCC; urine color chart, RPE: rating of perceived effort, ms: milliseconds, kg: kilograms, cm: centimeters.

## Data Availability

All data used in this research are in the manuscript.
